# Enhancement in electron transport and light emission efficiency of a Si nanocrystal light-emitting diode by a SiCN/SiC superlattice structure

**DOI:** 10.1186/1556-276X-8-14

**Published:** 2013-01-05

**Authors:** Chul Huh, Bong Kyu Kim, Byoung-Jun Park, Eun-Hye Jang, Sang-Hyeob Kim

**Affiliations:** 1 IT Convergence Technology Research Laboratory, Electronics and Telecommunications Research Institute, Daejeon 305-350, Republic of Korea

**Keywords:** Light-emitting diode, Si nanocrystal, Superlattice, Electron transport, Wall-plug efficiency

## Abstract

We report an enhancement in light emission efficiency of Si nanocrystal (NC) light-emitting diodes (LEDs) by employing 5.5 periods of SiCN/SiC superlattices (SLs). SiCN and SiC layers in SiCN/SiC SLs were designed by considering the optical bandgap to induce the uniform electron sheet parallel to the SL planes. The electrical property of Si NC LED with SiCN/SiC SLs was improved. In addition, light output power and wall-plug efficiency of the Si NC LED with SiCN/SiC SLs were also enhanced by 50% and 40%, respectively. This was attributed to both the formation of two-dimensional electron gas, i.e., uniform electron sheet parallel to the SiCN/SiC SL planes due to the conduction band offset between the SiCN layer and SiC layer, and an enhanced electron transport into the Si NCs due to a lower tunneling barrier height. We show here that the use of the SiCN/SiC SL structure can be very useful in realizing a highly efficient Si NC LED.

## Background

Until now, lots of research have been devoted towards the development of Si-based light sources that could enable the integration of photonics with Si microelectronics [1-3]. Si-based light sources could reduce the fabrication cost because their compatibility with a conventional complementary metal-oxide semiconductor (CMOS) technology is better than any other light source such as conventional GaAs- and GaN-based light emitters. Despite a lot of efforts for the realization of Si-based light sources with high efficiency, luminescence efficiency from Si-based light sources is still very low due to an indirect bandgap nature of the bulk Si [4,5]. Recently, because of this obstacle for realizing efficient Si-based light sources, Si nanocrystals (NCs) have, therefore, attracted the most attention as promising light sources for the next generation of Si-based nanophotonics [6-8]. Si NCs showed a quantum confinement effect that increased in the overlapping of electron–hole wave functions, leading to an enhancement in luminescence efficiency [9]. Another advantage for light sources using Si NCs is that the optical bandgap can be easily tuned by changing the size of NCs. This implies that Si NCs are of particular interest as a light source, covering the whole visible wavelength range.

Si NCs have been generally synthesized into insulating matrices. Because of this, disadvantages appear in realizing an efficient Si NC light-emitting diode (LED). To realize efficient Si NC LEDs, therefore, following required factors such as the formation of Si NCs with high density, surrounding matrix, and design of an efficient carrier injection film should be addressed. We and others have recently demonstrated an *in situ* growth of well-organized Si NCs in a Si nitride (SiN_*x*_) matrix by conventional plasma-enhanced chemical vapor deposition (PECVD) and have achieved a reliable and stable tuning of the wavelength ranging from near infrared to ultraviolet by changing the size of Si NCs [8,10,11]. SiN_*x*_ as a surrounding matrix for Si NCs can provide advantages over generally used Si oxide films because of the *in situ* formation of Si NCs at low temperature, small bandgap, and clear quantum confinement dependence on the size of Si NCs. These merits can meet the requirements for the current CMOS technology such as compatibility with integration and cost-effectiveness. To inject the carriers into the Si NCs, polysilicon, indium tin oxide (ITO), and semitransparent metal films have been generally used as contact materials [12-14]. However, the photons generated from the Si NCs could be absorbed because the photons passed through these contact materials to escape out from the Si NC LEDs. A suitable carrier injection layer is, therefore, very crucial for enhancing the light emission efficiency of Si NC LEDs. In previous results [15,16], we grew the amorphous SiC(N) film with an electron density up to 10^19^ cm^−3^ using a PECVD at 300°C and demonstrated that the amorphous SiC(N) film could be a suitable electron injection layer to improve the light emission efficiency of Si NC LEDs.

Recently, alternative methods such as surface plasmons (SPs) by nanoporous Au film [17] or Ag particles [18] that could enhance the luminescence efficiency from the Si NCs and external quantum efficiency of a Si quantum dot LED were reported. These approaches, however, need complicated wet etching and annealing processes to apply SP coupling. They also have disadvantages in realizing an efficient Si NC LED, such as having an impractical structure for LED fabrication and absorption of light escaping out from the LED at the metal layer. A reliable, simple, and practical device design without additional processes is, hence, very crucial in the fabrication and an enhancement of the light emission efficiency of Si NC LED. In this work, we present the concept that can uniformly transport the electrons into the Si NCs by employing 5.5 periods of SiCN/SiC superlattices (SLs) specially designed for an efficient electron transport layer, leading to an enhancement in the light emission efficiency of Si NC LED. A SiCN film in 5.5 periods of SiCN/SiC SLs was designed to have a higher optical bandgap than that of SiC to induce a two-dimensional electron gas (2-DEG), i.e., uniform electron sheet, at the interface between the SiCN and SiC layers due to the conduction band offset between these two layers. The electrical characteristic of Si NC LED with the SLs was improved. Moreover, light emission efficiency and wall-plug efficiency (WPE) of the Si NC LED with the SLs were also enhanced by 50% and 40%, respectively.

## Methods

The Si NCs used here were embedded into a SiN_*x*_ matrix with a thickness of 50 nm and were *in situ* grown by PECVD, in which Ar-diluted 10% SiH_4_ and NH_3_ was used as the source of reactants. The plasma power, chamber pressure, and substrate temperature for the growth of Si NCs were fixed at 5 W, 500 mTorr, and 250°C, respectively. The size of Si NCs embedded into a SiN_*x*_ was around 4 nm, which was confirmed by high-resolution transmission electron microscopy (HRTEM) [10]. No post annealing process was performed to create the Si NCs into the SiN_*x*_ matrix after the growth. SiCN (3 nm)/SiC (3 nm) SLs at 5.5 periods doped with phosphorous (P) was deposited on the Si NCs which were embedded into the SiN_*x*_ matrix at 300°C by a PECVD. The SiCN/SiC SLs were grown by changing the flow rates of CH_4_ and NH_3_ sources while the flow rate of SiH_4_ was fixed. An amorphous SiC film (approximately 40 nm) doped with P that is used as an electron injection layer was deposited on the 5.5 periods of SiCN/SiC SLs. An ITO layer (100 nm) used as a transparent current spreading layer was deposited at 150°C on an amorphous SiC film and then annealed at 300°C for 30 min in a pulsed laser deposition chamber to improve the electrical property and optical transparency. Right after the deposition of ITO, the Si NC LED samples were etched using an inductively coupled SF_6_/O_2_ plasma and standard photolithographic technique until the Si layer was exposed. Finally, a Ni/Au (30/120 nm) layer was deposited for the top and backside contacts using thermal evaporation. A mesa-type Si NC LED with 5.5 periods of SiCN/SiC SLs with an area of 300 × 300 μm^2^ was fabricated, and Si NC LED without SiCN/SiC SLs was also fabricated for comparison.

## Results and discussion

**Figure 1:**
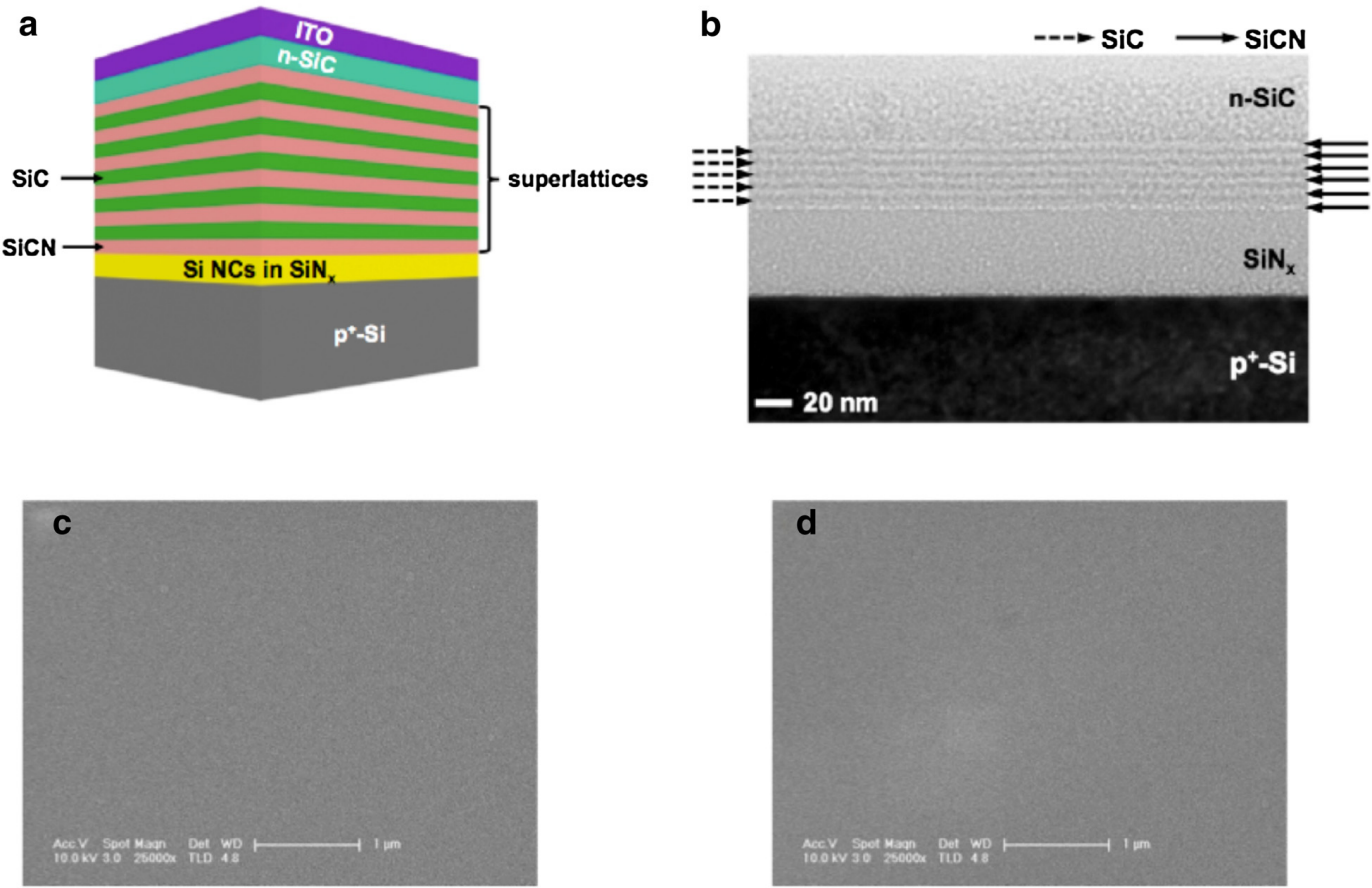
**Schematic illustration**,**HRTEM image**,**and SEM images.** (**a**) A schematic illustration of the Si NC LED with 5.5 periods of SiCN/SiC SLs. (**b**) An HRTEM image of Si NC LED with 5.5 periods of SiCN/SiC SLs. The interfaces between each layer of Si NC LED with the SLs were flat and abrupt. (**c**) SEM image of the SiC layer surface. (d) SEM image of the SiCN layer surface.

Figure 1a shows a schematic illustration of the Si NC LED with 5.5 periods of SiCN/SiC SLs. The SiCN/SiC SLs were designed by considering the optical bandgap to increase the electron injection into the Si NCs due to the formation of 2-DEG at the interface between the SiCN layer and SiC layer. Since SiN has a higher bandgap than SiC, the optical bandgap of the SiCN layer can be tuned by changing the N composition. By increasing the N composition in the SiCN layer, the optical bandgap would be increased. A higher optical bandgap has an advantage for enhancing the light extraction efficiency of Si NC LED since the photons generated in the Si NC layer can easily escape outside the LED by decreasing the absorption of photons at the SLs. In the previous result [16], however, we found that the SiCN layer showed an insulating property when the N composition in the SiCN layer exceeded over 20%. Hence, both the optical bandgap and electrical property of each layer in the SLs should be optimized to improve the performance of the Si NC LED. The N composition in the SiCN layer used in the SLs was about 18%. The optical bandgaps were determined from optical transmittance measurements of the films that were grown on quartz substrate by applying the Tauc model [19]. The optical bandgaps of the SiCN and SiC layers in the SLs were estimated to be around 2.6 and 2.2 eV, respectively. The electron densities of the SiCN and SiC layers were measured at room temperature using the Hall measurement system and were determined to be 4 × 10^18^ and 2 × 10^17^ cm^−3^, respectively. The electron density of the SiCN layer was 20 times higher than that of the SiC layer. Figure 1b shows the HRTEM image of the Si NC LED with 5.5 periods of SiCN/SiC SLs. The interfaces between the SiCN and SiC layers consisting the SLs were flat and abrupt, suggesting that the structural property of the 5.5 periods of SiCN/SiC SLs was quite good. Figure 1c,d shows the SEM images of the surfaces of the SiC and SiCN layers, respectively. As shown in Figure 1c,d, the surfaces of the SiC and SiCN layers were very smooth.

**Figure 2:**
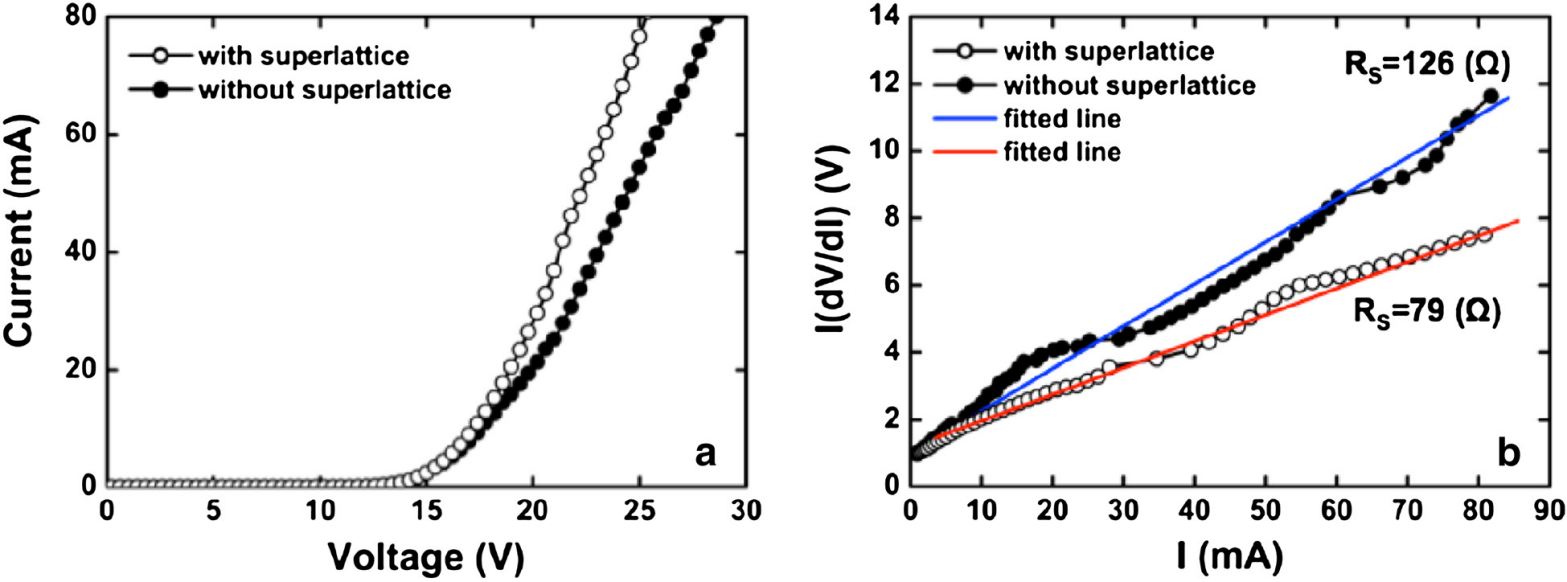
***I***-***V *curves and series resistances of Si NC LEDs.** (**a**) *I*-*V* curves of Si NC LEDs with and without 5.5 periods of SiCN/SiC SLs, respectively. (**b**) Series resistances of Si NC LEDs with and without 5.5 periods of SiCN/SiC SLs, respectively.

The current–voltage (*IV*) curves of Si NC LED with and without 5.5 periods of SiCN/SiC SLs measured at room temperature, respectively, are shown in Figure 2a. The *IV* curve of Si NC LED with 5.5 periods of SiCN/SiC SLs was better than that of Si NC LED without the SLs, as can be clearly seen in Figure 2a. In order to investigate the effect of SLs on the electrical property of Si NC LED, the typical on-series resistance (*R*_*S*_) of Si NC LEDs with and without 5.5 periods of SiCN/SiC SLs was calculated using the measured *IV* curves shown in Figure 2a. The *R*_*S*_ was calculated from the diode relation of a p-n junction. When the *R*_*S*_ contributes to device behavior, the diode equation can be written as $I=I_{0}\left(e^{q\left(V-IR_{S}\right)/\mathrm{nkT}}-1\right)$, where *I*_0_ is the prefactor, *V* is the measured voltage, and *n* is the ideality factor [20]. This equation can be rewritten as *I*(*dV*/*dI*) = *IR*_*S*_ + *nkT*/*q*, indicating that *R*_*S*_ and *n* can be extracted from the slope and *y*-axis intercept of this equation. The *R*_*S*_ values were calculated to be 126 and 79 Ω, respectively, as shown in Figure 2b. The *R*_*S*_ for Si NC LED with 5.5 periods of SiCN/SiC SLs significantly decreased as compared with that of Si NC LED without 5.5 periods of SiCN/SiC SLs.

**Figure 3:**
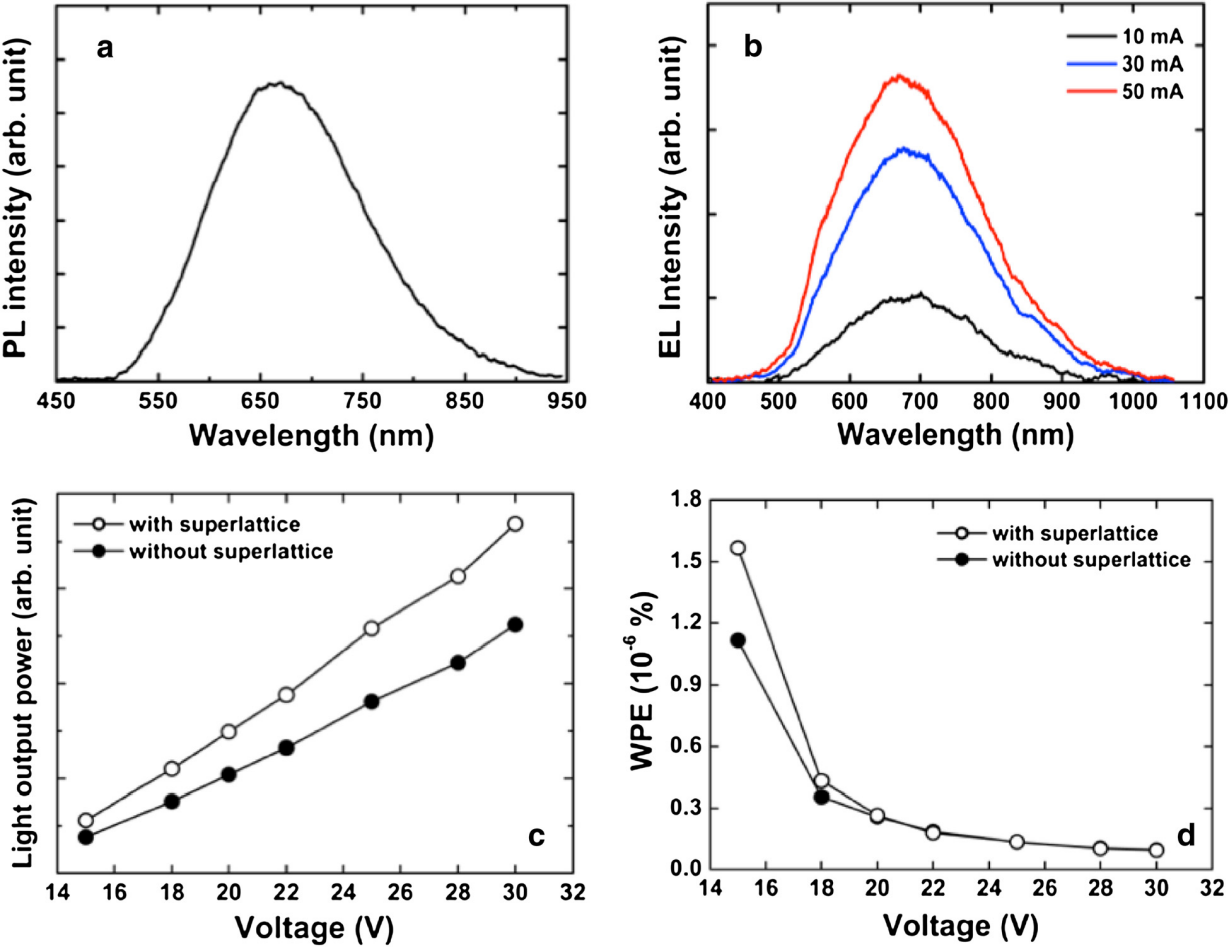
**PL**,**EL**,**light output powers**,**and WPEs.** (**a**) PL spectrum taken from the Si NCs in the SiN_*x*_. The main peak position was around 680 nm. (**b**) EL spectra taken from the Si NC LED with 5.5 periods of SiCN/SiC SLs. The main peak position was around 680 nm. (**c**) Light output powers of Si NC LEDs with and without 5.5 periods of SiCN/SiC SLs, respectively. (**d**) WPEs of Si NC LEDs with and without 5.5 periods of SiCN/SiC SLs, respectively.

Figure 3a shows a photoluminescence (PL) spectrum taken from the Si NCs in the SiN_*x*_. A charge-coupled device detector was employed for the PL measurement at room temperature, with an He-Cd 325-nm laser as the excitation source. The main peak position was around 680 nm. The electroluminescence (EL) spectra were taken from the Si NC LED with 5.5 periods of SiCN/SiC SLs as a function of forward current, which was measured at room temperature, as shown in Figure 3b. Both PL and EL showed a similar center peak position at 680 nm. This indicates that the PL and EL processes can be related to the same luminescence mechanism that originated from the Si NCs. As shown in Figure 3b, the EL intensity increased with the increasing forward current. Figure 3c shows the light output powers of Si NC LEDs with and without 5.5 periods of SiCN/SiC SLs, which were measured at room temperature, respectively. Light output power of the Si NC LEDs was measured through the top side of the Si NC LEDs at a single wavelength using a Si photodiode connected to an optical power meter (Newport 818-SL), not from integrated measurement, because the total light output power from the Si NC LEDs is very difficult to measure or calculate without a packaging. Light output power of the Si NC LED with 5.5 periods of SiCN/SiC SLs improved by 50% compared with that of the Si NC LED without the SLs, as can be seen in Figure 3c. The power efficiency (output power/input power) is very important in real LED applications to reduce power consumption. The wall-plug efficiencies (WPEs), as shown in Figure 3d, were calculated based on the *IV* data and light output power. The WPEs of Si NC LEDs with and without 5.5 periods of SiCN/SiC SLs were estimated to be 1.06 and 1.57 × 10^−6^% at an input voltage of 15 V, respectively. The WPE of Si NC LED with 5.5 periods of SiCN/SiC SLs increased by 40% compared with that of the Si NC LED without the SLs. With increasing input voltage, WPEs of the Si NC LEDs with and without the SLs decreased, as shown in Figure 3d. The WPEs of Si NC LEDs with and without the SLs have similar values over the input voltage of 20 V. Increasing the input voltage means that the input current injected into the Si NC LED increases. Despite the increase in the current injected into the Si NC LED, decreasing the WPE suggests that the current injected into the Si NC LED would not efficiently transport into the Si NCs. This indicates that the increase in light output power as the current was increased was not enough. This result could be attributed to the defects in the SiN_*x*_ used as the surrounding matrix. Since the SiN_*x*_ contained Si NCs in the amorphous phase, more defects such as vacancies and dislocations could be created compared with the crystalline phase. Therefore, the current injected into the Si NC LED was not efficiently transported into the Si NCs but passed through the defects, resulting in the recombination of electron–hole pairs as the Si NCs decreased. Another reason for decreasing the WPEs with the increasing current can be due to the hot electrons. With the increasing input power, the electrons injected into the Si NC layer are more energetic due to higher electric field. As a result, the hot electrons could pass through the SiN_*x*_ without recombining at the Si NCs, resulting in the decrease in output power, i.e., WPE. This phenomenon would be depressed if the defects in the SiN_*x*_ will be decreased through the growth optimization of the surrounding SiN_*x*_ matrix. An alternative possibility for enhancing the recombination efficiency of electron–hole pairs at the Si NCs could be the design of the luminescent layer containing the Si NCs such as the multi-quantum well structure or electron blocking layer for preventing electron overflow from the luminescent layer generally used in organic, GaN-, and GaAs-based LEDs [21-24]. Based on the results of light output power and WPE, as can be seen in Figure 3c,d, use of the SL structure is a crucial role in enhancing the light output power and WPE of the Si NC LED.

**Figure 4:**
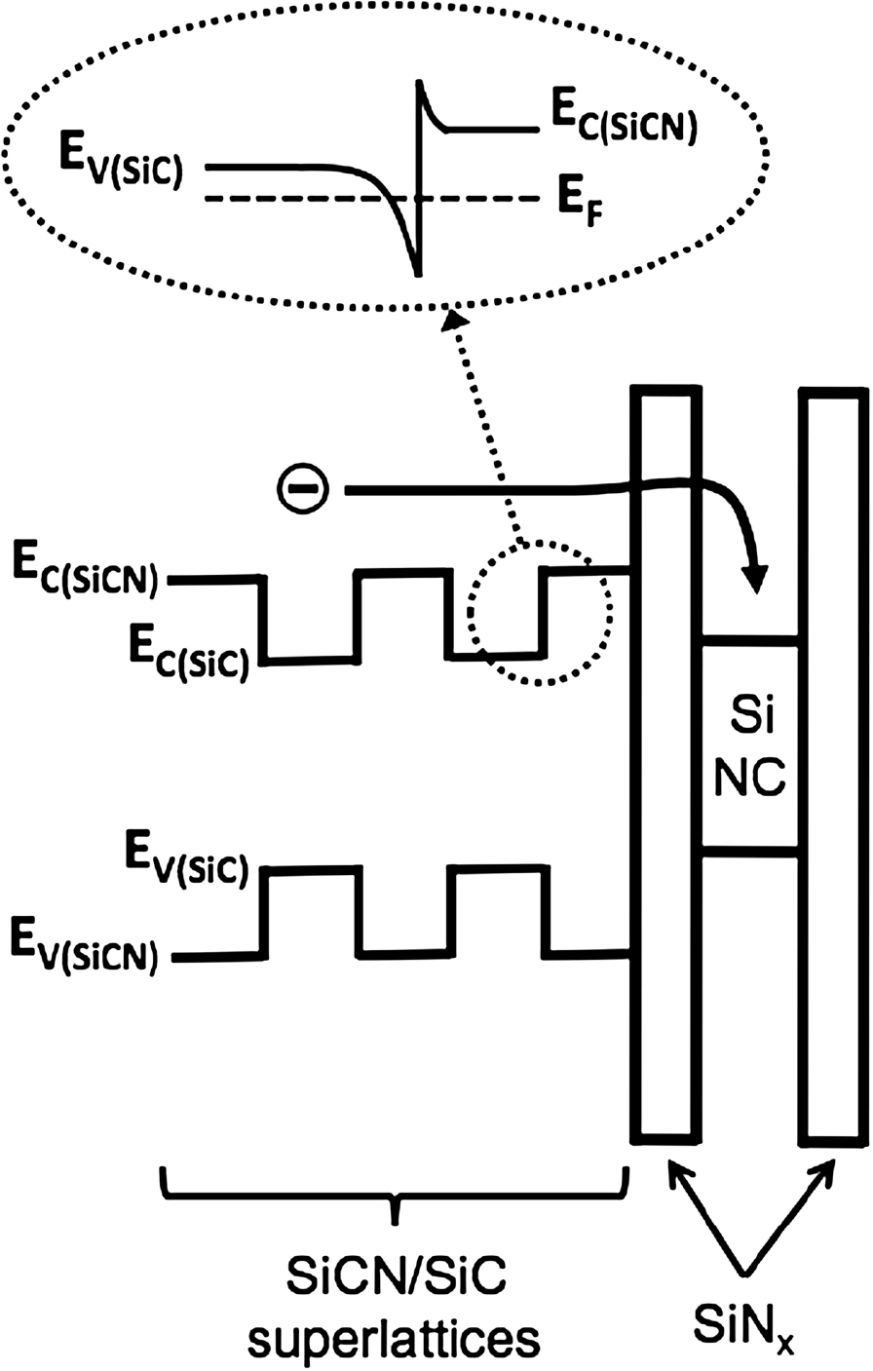
**A schematic band diagram of the Si NC LED with 5**.**5 periods of SiCN**/**SiC SLs.** A dotted oval in the upper part shows a specific conduction band diagram at the interface between SiCN and SiC layers in the SLs showing the formation of 2-DEG.

Figure 4 shows a schematic bandgap diagram of the Si NC LED with 5.5 periods of SiCN/SiC SLs. A dashed oval in the upper part of Figure 4 shows a conduction band diagram at the interface between SiCN and SiC layers in the SLs showing the formation of 2-DEG. It is generally known that the SLs are widely used to enhance the carrier transport to the active layer [25,26]. By assuming the band offset (Δ*E*) to be half the difference in the bandgaps of the SiCN (2.6 eV) and SiC (2.2 eV) layers, the conduction band offset (Δ*E*_c_) is 200 meV since the total band offset is 400 meV. Because of this Δ*E*_c_, the 2-DEG, i.e., uniform electron sheet, can be formed along the lateral direction of the SiC layer to coincide the Fermi level of the SiCN and SiC layers. Another important thing is the lowering of the tunneling barrier height for electrons to transport into the Si NCs. For the SiCN layer, the electrons have a lower tunneling barrier by 200 meV due to the higher bandgap, as can be seen Figure 4. These indicates that the electrons can be efficiently transported into Si NCs through the overlaying SiCN layer compared to the SiC layer, resulting in an increase in the light emission efficiency. Therefore, the improved electrical property and enhanced light output power of Si NC LED with the SiCN/SiC SLs, as shown in Figures 2 and 3c, were attributed to the formation of uniform electron sheet parallel to the SL planes due to the Δ*E*_c_ and an enhancement in electron transport into Si NCs due to the lower tunneling barrier height.

## Conclusions

We demonstrate the fabrication of Si NC LED with 5.5 periods of SiCN/SiC SLs. SiCN/SiC SLs at 5.5 periods was designed by considering the optical bandgap to form the uniform electron sheet parallel to the SL planes. The electrical property of Si NC LED with 5.5 periods of SiCN/SiC SLs was improved. Moreover, light output power and WPE of the LED with 5.5 periods of SiCN/SiC SLs were also enhanced by 50% and 40%, respectively, which were ascribed to the formation of uniform electron sheet and enhancement in electron transport in Si NCs. We show here that the SiCN/SiC SL structure can be used to realize a highly efficient Si NC LED.

## Competing interests

The authors declare that they have no competing interests.

## Authors’ contributions

CH grew the Si NC samples, fabricated LEDs, and performed the HRTEM analysis. CH also conceived the study, participated in its design and coordination, and drafted the manuscript. BKK participated in measuring the electrical characteristics and their corresponding analysis. BJP performed the PL measurement. EHJ participated in measuring the EL spectra. SHK participated in measuring the optical properties. All authors read and approved the final manuscript.
